# Seasonal Variation in the Brain μ-Opioid Receptor Availability

**DOI:** 10.1523/JNEUROSCI.2380-20.2020

**Published:** 2021-02-10

**Authors:** Lihua Sun, Jing Tang, Heidi Liljenbäck, Aake Honkaniemi, Jenni Virta, Janne Isojärvi, Tomi Karjalainen, Tatu Kantonen, Pirjo Nuutila, Jarmo Hietala, Valtteri Kaasinen, Kari Kalliokoski, Jussi Hirvonen, Harry Scheinin, Semi Helin, Kim Eerola, Eriika Savontaus, Emrah Yatkin, Juha O. Rinne, Anne Roivainen, Lauri Nummenmaa

**Affiliations:** ^1^Turku PET Centre, University of Turku, FIN-20520 Turku, Finland; ^2^Research Program in Systems Oncology, Faculty of Medicine, University of Helsinki, FIN-00014 Helsinki, Finland; ^3^Turku Center for Disease Modeling, University of Turku, FIN-20520 Turku, Finland; ^4^Turku PET Centre, Turku University Hospital, FIN-20520, Turku, Finland; ^5^Clinical Neurosciences, Turku University Hospital, FIN-20520 Turku, Finland; ^6^Department of Endocrinology, Turku University Hospital, FIN-20520 Turku, Finland; ^7^Department of Psychiatry, Turku University Hospital, FIN-20520 Turku, Finland; ^8^Department of Radiology, University of Turku, FIN-20520 Turku, Finland; ^9^Research Unit for Integrative Physiology and Pharmacology, Institute of Biomedicine, University of Turku, FIN-20520 Turku, Finland; ^10^Unit of Clinical Pharmacology, Turku University Hospital, FIN-20520 Turku, Finland; ^11^Central Animal Laboratory, University of Turku, Turku, FIN-20520, Finland; ^12^Department of Psychology, University of Turku, FIN-20520 Turku, Finland

**Keywords:** brain, emotion, μ-opioid receptor, neurotransmission, PET, seasonal affective changes

## Abstract

Seasonal rhythms influence mood and sociability. The brain μ-opioid receptor (MOR) system modulates a multitude of seasonally varying socioemotional functions, but its seasonal variation remains elusive with no previously reported *in vivo* evidence. Here, we first conducted a cross-sectional study with previously acquired human [^11^C]carfentanil PET imaging data (132 male and 72 female healthy subjects) to test whether there is seasonal variation in MOR availability. We then investigated experimentally whether seasonal variation in daylength causally influences brain MOR availability in rats. Rats (six male and three female rats) underwent daylength cycle simulating seasonal changes; control animals (two male and one female rats) were kept under constant daylength. Animals were scanned repeatedly with [^11^C]carfentanil PET imaging. Seasonally varying daylength had an inverted U-shaped functional relationship with brain MOR availability in humans. Brain regions sensitive to daylength spanned the socioemotional brain circuits, where MOR availability peaked during spring. In rats, MOR availabilities in the brain neocortex, thalamus, and striatum peaked at intermediate daylength. Varying daylength also affected the weight gain and stress hormone levels. We conclude that cerebral MOR availability in humans and rats shows significant seasonal variation, which is predominately associated with seasonal photoperiodic variation. Given the intimate links between MOR signaling and socioemotional behavior, these results suggest that the MOR system might underlie seasonal variation in human mood and social behavior.

**SIGNIFICANCE STATEMENT** Seasonal rhythms influence emotion and sociability. The central μ-opioid receptor (MOR) system modulates numerous seasonally varying socioemotional functions, but its seasonal variation remains elusive. Here we used positron emission tomography to show that MOR levels in both human and rat brains show daylength-dependent seasonal variation. The highest MOR availability was observed at intermediate daylengths. Given the intimate links between MOR signaling and socioemotional behavior, these results suggest that the MOR system might underlie seasonal variation in human mood and social behavior.

## Abstract (precis)

Seasonal rhythms influence mood and sociability. The brain μ-opioid receptor (MOR) system modulates a multitude of seasonally varying socioemotional functions, but its seasonal variation remains elusive with no previously reported *in vivo* evidence. Here, we first conducted a cross-sectional study with previously acquired human [^11^C]carfentanil PET imaging data (132 male and 72 female healthy subjects) to test whether there is seasonal variation in MOR availability.

## Introduction

Seasonal rhythms profoundly impact mood. Negative affect including depression, anger, and hostility is at lowest during the summer (Harmatz et al., 2000), whereas seasonal affective disorder rates peak during the winter months (Lam and Levitan, 2000). These changes are mediated by slow phasic changes in different neuroreceptor systems. For instance, long daylength increases brain serotonin and norepinephrine levels in mice and reduces depression and anxiety behavior, compared with short daylength (Green et al., 2015). The duration of daylight exposure is similarly associated with brain serotonin turnover in humans (Lambert et al., 2002). The intimate link between seasonal fluctuations in mood and the contribution of opioidergic neurotransmission in human emotions (Nummenmaa and Tuominen, 2018; Nummenmaa et al., 2020) suggests potential seasonal variation of *in vivo* μ-opioid receptor (MOR) signaling.

Several lines of evidence suggest that MOR availability could vary seasonally in humans. Opioids are among the most commonly used illicit drugs in the United States, where 2% of the population have had opioid use disorder during their lifetime (Tung et al., 2018). Suicidal behavior with prescription opioids follows a clear seasonal pattern, with suicide attempts peaking in the spring and fall (Davis et al., 2014). In contrast, the overall number of opioid overdose deaths (Sadler and Furr-Holden, 2019) and the amount of city wastewater opioid metabolites (Krizman-Matasic et al., 2019) is the lowest in summer. Further, postmortem studies have established that suicide victims have increased MOR densities (Gross-Isseroff et al., 1990; Gabilondo et al., 1995). Although these studies have not directly assessed seasonal effects, it is well established that suicide rates peak in the spring regardless of the geographical location of the country (Woo et al., 2012). Finally, MORs are potent modulators of feeding (Karlsson et al., 2015; Tuulari et al., 2017; Nummenmaa et al., 2018) because of their contribution of hedonic or “liking” responses in the brain (Berridge, 2009), and human feeding patterns show seasonal variation, with caloric intake of fats typically peaking in the fall or winter (Shahar et al., 1999; Ma et al., 2006). However, direct *in vivo* evidence on the causal seasonal effects on opioidergic neurotransmission is currently lacking.

Animal studies also suggest that there is seasonal variation in MOR-dependent signaling. The endogenous opioid signaling is crucial for the photoperiodic control of the seasonal reproductive cycle in mammals (Tortonese, 1999). Endogenous opioids inhibit the release of gonadotropin and sex hormones, and this inhibitory effect is enhanced under short daylength compared with long daylength (Lincoln et al., 1987). Also, MOR expression in hamster testes is increased during short days, with increased inhibitory control for testosterone secretion (Mukherjee and Haldar, 2016). In Siberian hamsters, brain expression of dynorphin A, an endogenous peptide agonist for opioid receptors, is increased under longer versus shorter daylength (Meyza and Sotowska-Brochocka, 2006), suggesting a potential impact of daylength on opioidergic signaling in the brain. Furthermore, the effects of morphine on the feeding behavior of ground squirrels vary in accord with the hibernation state (Nizielski et al., 1986), indirectly reflecting a seasonal variation of MOR signaling.

Here, we tested whether the seasonal variation in daylength, a key determinant of seasonal rhythms, influences MOR availability in the brain. MOR availability was quantified using *in vivo* positron emission tomography (PET) with the MOR-sensitive agonist ligand [^11^C]carfentanil. We first conducted a cross-sectional study with previously acquired human PET imaging data to test whether there are seasonal differences in MOR availability. Considering the relationship among daylight exposure, stress, and brain functions (Vondrasová et al., 1997; Sapolsky, 2015), we anticipated that both linear and inverted-U functional relationship between daylength and brain MOR availability were possible. We then investigated experimentally whether seasonal variation in daylength causally influences brain MOR availability in rats. While rats underwent daylength cycle simulating seasonal changes, repeated [^11^C]carfentanil PET imaging was conducted. Our data suggest that there is seasonal variation in brain MOR availability in both humans and rats, with MOR availability peaking in seasons with intermediate daylengths.

## Materials and Methods

### 

#### Human PET imaging study

##### Subjects

**Figure 1.**
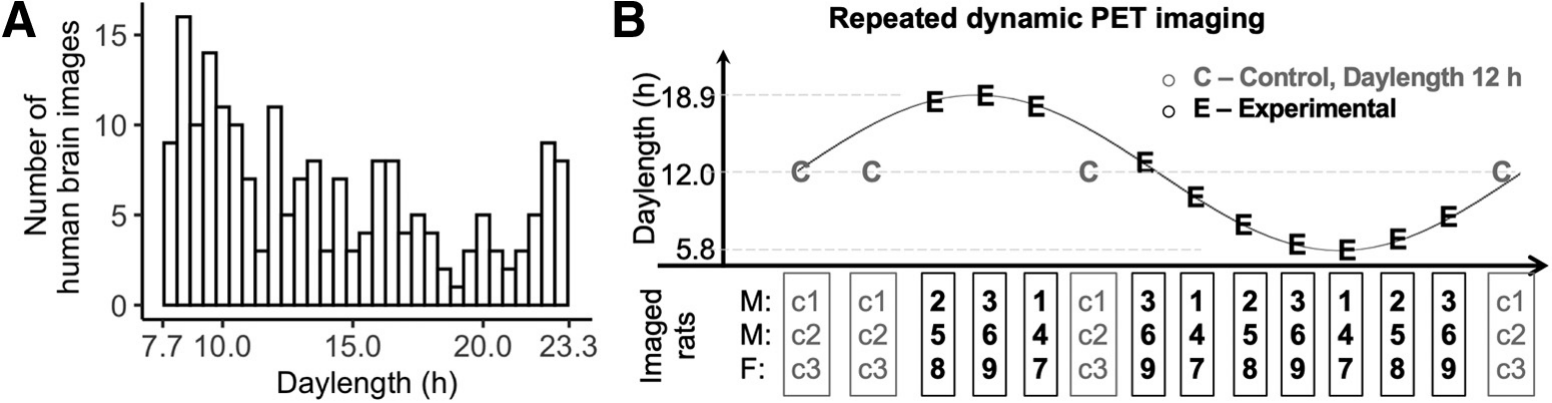
Study design. ***A***, Number of human PET scans at different daylengths (1 bin = 0.5 h). ***B***, Experimental design for the rat model PET study. M, Males; F, females.

Human study was performed using retrospective analysis of historical subjects scanned with PET using radioligand [^11^C]carfentanil at different times of the year (Fig. 1). The data were retrieved from the AIVO database (https://aivo.utu.fi) of *in vivo* molecular images hosted by Turku PET Centre. We identified all the baseline scans from individuals with no neurologic or psychiatric disorders who had been scanned between 2003 and 2018. The resulting data frame of 204 individuals (132 males, 72 females; mean age, 32.4 ± 10.8 years) consists of scans from 11 research projects and five PET scanners, with details of the subjects described in Kantonen et al. (2020).

##### PET image analysis

PET data were analyzed and modeled using the Magia toolbox (https://github.com/tkkarjal/magia; Karjalainen et al., 2020), an automated processing pipeline developed at the Turku PET Centre running on MATLAB (MathWorks). Preprocessing consisted of framewise realignment and coregistration of the PET and magnetic resonance images. Tracer binding was quantified using *BP*_ND_, which is the ratio of specific binding to nondisplaceable binding in tissue. *BP*_ND_ was estimated using a simplified reference tissue model with occipital cortex as the reference region. Parametric *BP*_ND_ images were also calculated for full-volume analysis. They were spatially normalized to MNI space via segmentation of T1-weighted MRIs and smoothed with an 8 mm Gaussian kernel. Regions of interest (ROIs), including the reference region, were parcellated for each subject using FreeSurfer (https://surfer.nmr.mgh.harvard.edu/).

##### Full-volume human data analysis

For each subject, daylength was calculated as the daytime plus civil twilight on the day when the PET image was acquired. Civil twilight comprises of morning civil twilight, which begins when the geometric center of the sun is 6° below the horizon and ends at sunrise, and evening civil twilight, which begins at sunset and ends when the geometric center of the sun reaches 6° below the horizon. Calculation was performed using the R package “suncalc,” where calculations were based on geographic location of the Turku PET Center (Turku, Finland; latitude = 60.4518; longitude = −22.2666).

We first modeled the effect of daylength on MOR availability using multiple regression, as implemented in SPM12 (http://www.fil.ion.ucl.ac.uk/spm/). Polynomial expansion of daylength to the second order and the linear component of daylength were used as regressors. Subject age, sex, scanner, and body mass index (BMI) were used as nuisance covariates. MOR *BP*_ND_ values in the daylength-sensitive brain cluster were also extracted using the MarsBaR toolbox and were plotted as a function of daylength and seasons for visualization.

##### Region of interest analysis

We also analyzed the regional MOR availability in 15 brain ROIs (based on subject-wise FreeSurfer delineations) for both hemispheres (in total, 30 ROIs), as listed in Table 2. These regions were selected because they have high MOR binding, and they also contribute significantly to emotion and mood regulation in humans (Saarimäki et al., 2016; Karjalainen et al., 2017; Nummenmaa and Tuominen, 2018). Regional MOR *BP*_ND_ was log transformed in the statistical analysis as previously described (Kantonen et al., 2020). Pooled ROI values were analyzed using a linear mixed-effects model with varying intercepts for each subject. Fixed-effect factors included daylength, squared daylength, age, sex, BMI, scanner type, and their interaction effects with ROI and brain hemisphere (right vs right). R statistical software (version 3.6.0) using the lme4 package was used in analyzing the human ROI data and the rat data.

**Table 1.** Type III analysis of variance with Satterthwaite's method for the human data region of interest analysis

Factors	All		Male (*n* = 132)		Female (*n* = 72)	
	Mean of squares	Percentage (%)	Mean of squares	Percentage (%)	Mean of squares	Percentage (%)
Daylength	0.045	2.29	0.033	2.66	0.013	1.38
Squared daylength	0.044	2.26	0.035	2.82	0.012	1.28
Age	0.061	3.11	0.073	5.93	0.005	0.52
Scanner	0.070	3.56	0.050	4.10	0.000	0.000
BMI	0.002	0.09	0.001	0.11	0.007	0.76
Sex	0.009	0.48				

The mean of squares and the percentage of contribution to the full model for daylength, squared daylength, age, scanner, BMI, and sex are listed. We also listed these values for each sex separately.

#### PET imaging study with rats

##### Animal handling and seasonal simulation

The National Animal Experiment Board of Finland approved all the procedures and protocols (license #ESAVI/8355/2019) in accordance with the EU Directive 2010/63/EU on the protection of animals used for scientific purposes. Eighteen adult Sprague Dawley rats (age, >90 d; 11 males, 7 females; Central Animal Laboratory, University of Turku, Turku, Finland; N.B., 12 rats underwent PET imaging, as described in the next section) were housed under controlled laboratory conditions in open top cages with free access to CRM-E diet (SDS) and water. Rats were caged in groups of two or three same-sex rats. No rats were removed before finishing the last PET imaging to keep the social environment stable and to avoid additional stress (Beery and Kaufer, 2015). For the rats in the experimental group (nine males, five females), in-house light with variable ON/OFF duration was programmed, simulating the local seasonal change of daylength with a speeded cycle completed in 3 months (Fig. 1). Rats in the control group (two males, two females) were kept in a different room with a constant daylength cycle (12 h ON/12 h OFF, no twilight), with all other conditions the same as with the experimental group.

The control group was used for addressing the potential effects of aging and weight gain on MOR levels that occur in the absence of variable daylength; the main statistical analyses pertaining to MOR were also run separately in the experimental group only. The same type of LED lighting was used for control groups and the experimental groups.

##### PET imaging and processing

Twelve of 18 rats (experimental group: 6 males, 3 females; control group: 2 males, 1 female) were studied with dynamic [^11^C]carfentanil PET imaging three to four times under isoflurane anesthesia. Radiotracers were divided by three rats at each scanning day (two males, one female), and via using two different scanners to maximize the data collection. Because of larger body size, Inveon Multimodality PET/computed tomography (CT; Siemens Medical Solutions) was used for imaging the male rats (two rats each time), whereas PET/CT scanning (MOLECUBES) was used for the female rats. In total, there were 42 PET/CT scans. Rats were weighed on the scanning day. For the Inveon Multimodality PET/CT scanner, with relatively lower resolution and sensitivity, the aimed dosage of radiotracer was 5 MBq, while for the MOLECUBES PET/CT scanner rats were scanned with an aimed dosage of 1 MBq. Accordingly, sex effects cannot be separated from scanner type and dosage-dependent effects.

Dynamic PET images were analyzed using Carimas software (version 2.10.3.0) developed at the Turku PET Center. The PET datasets were reconstructed in 20 time frames using the OSEM3D algorithm, as follows: 6 × 0.5, 3 × 1, 4 × 3, and 7 × 6 min. Images were processed as follows: we first created a template with an aligned structural CT skull image and brain atlas. In the template, a skull CT image of a rat was aligned with the Waxholm Space Atlas of the Sprague Dawley rat brain, which was used as the reference for the normalization of the brain scans. Thereafter, each skull CT scan (aligned with and transformed together with the PET scan for following steps) was resized manually and then coregistered with the template CT skull image. Finally, the overlapping brain atlas and each PET scan were applied for extracting regional time–activity curves (TACs).

A simplified reference tissue model was used for estimating *BP*_ND_ in the neocortex, striatum, and thalamus (Innis et al., 2007). The cerebellum was used as the reference region because it is devoid of MOR in rats (see Fig. 4*C*, *ex vivo* biodistribution results). We excluded the first 5 min from model fitting to rule out confounding perfusion effects. TACs from brain regions of interest (thalamus, striatum, and neocortex) defined by the Waxholm Space Atlas were then extracted for data analysis.

##### Ex vivo gamma counting

Twenty minutes after the injection of [^11^C]carfentanil (40 MBq), rats were killed under isoflurane anesthesia, and samples of various tissues and brain regions were excised, weighed, and measured for radioactivity using a gamma counter (Triathler 3 inch, Hidex). The results are shown as the percentage of injected radioactivity dose per gram of tissue.

##### Serum sampling and measurement of corticosterone levels

Blood samples were collected by lateral tail vein puncture at 9:00–11:00 A.M. For experimental group rats, samples included collections conducted 1 d before the PET imaging (30 of 33 samples). For the control group, sample collections were randomly conducted. Altogether, 51 blood samples were obtained (18 for the control group). The samples were centrifuged at 7000 rpm for 1.5 min, and the serum was stored at −20°C. Levels of serum corticosterone were measured using an ELISA kit (Enzo Life Sciences).

## Results

### Daylength-associated brain MOR availability in humans

**Figure 2.**
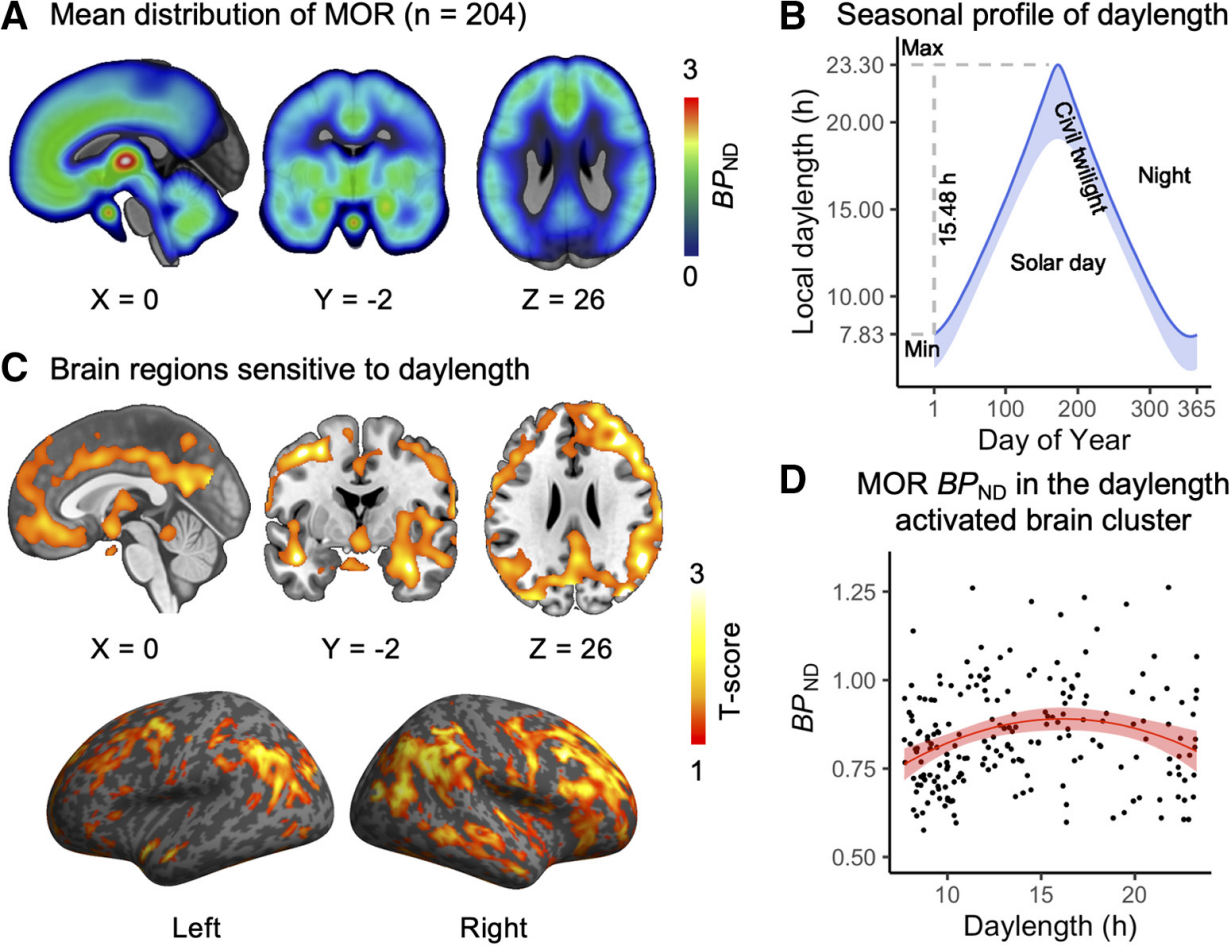
Human brain MOR availability and natural variation in daylength. ***A***, Mean distribution of MOR availability in the subjects. ***B***, Local seasonal distribution of daylength (day + civil twilight). ***C***, The brain cluster sensitive to the seasonal daylength changes in quadratic polynomial regression model. *p* < 0.05, FDR corrected. ***D***, [^11^C]carfentanil *BP*_ND_ in the brain cluster as a function of daylength. Red line shows polynomial LS (least-squares) regression curve (*y* ∼ *x* + *x*^2^) predicting *BP*_ND_, and shaded area shows 95% CI.

Figure 2*A* shows the mean MOR distribution in the human brain. We examined the association between natural variation in daylength (Fig. 2*B*) and MOR availability in humans. Brain full-volume analysis revealed a statistically significant quadratic polynomial effects in a large brain cluster (39,000 voxels; Fig. 2*C*,*D*), spanning the cingulate cortex; the superior frontal, medial frontal, middle temporal, and superior temporal gyrus; insula; and orbitofrontal cortex. Brain MOR availability in the brain cluster peaked at 15–19 h (Fig. 2*D*).

**Figure 3.**
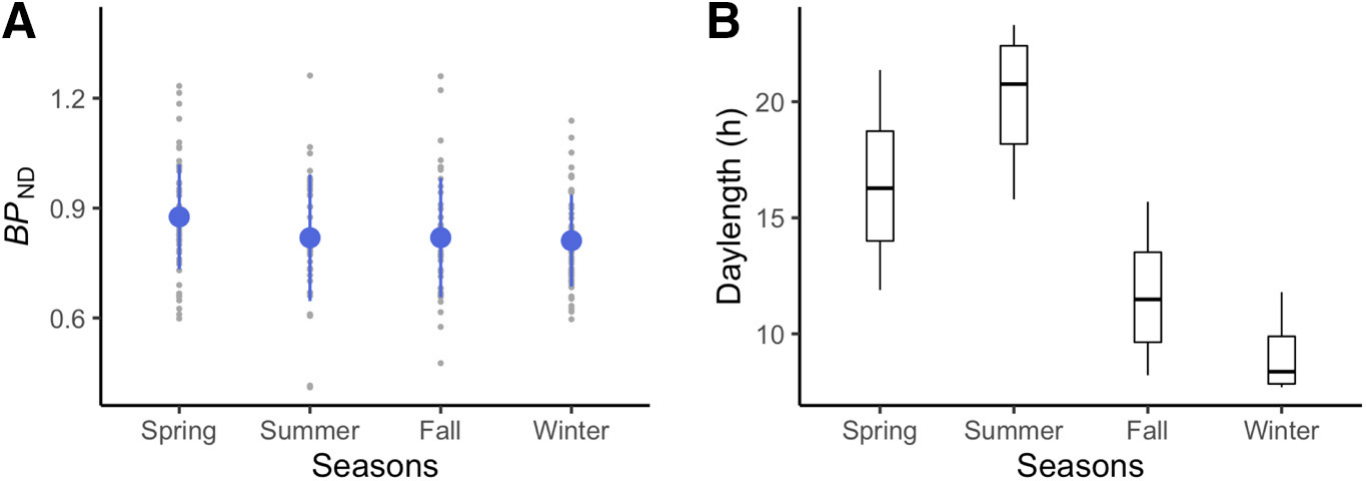
Visualization of season-dependent MOR availability as a function of meteorological seasons in humans. ***A***, Mean *BP*_ND_ in the cluster showing daylength-dependent variability in the full-volume analysis. Blue dots stand for means, and error bars stand for standard deviations. ***B***, Local daylength as a function of meteorological seasons.

Traditionally defined seasons, according to the northern meteorological definition, include spring (March, April, May), summer (June, July, August), fall (September, October, November), and winter (December, January, February). We plotted brain MOR *BP*_ND_ in the brain cluster in accord to local seasons for visualization (Fig. 3*A*). The plot shows that spring with a daylength of 16.39 ± 2.78 h (Fig. 3*B*) is associated with the highest MOR availability.

We next conducted region of interest analysis to assess specific regional effects of daylength on MOR binding. In the full model with all ROIs included, both daylength (β = 0.044; 95% CI, 0.008–0.08; *t* = 2.34) and squared daylength (β = −0.0014; 95% CI, −0.0026 to −0.00021; *t* = −2.30) were significant predictors for [^11^C]carfentanil *BP*_ND_, indicating an inverted U-shaped functional relationship. No interaction effects between daylength and squared daylength with brain hemisphere (left vs right) or ROIs were found. We also compared the effect of daylength on brain regional MOR availability with fixed-effect factors including age, sex, BMI, and scanner type by variance analysis of the model (Table 1). The effect of daylength on individual ROIs is shown in Table 2.

**Table 2.** Effect of daylength and squared daylength on regional MOR availability in the human brain (uncorrected for multiple comparison)

Hem	Region	Daylength			Squared daylength			F_(9, 194)_	Adj.*R*2
		β	95% CI	*t*	β	95% CI	*t*		
right	Amygdala	0.03	−0.005 to 0.07	1.71	−0.001	−0.002 to 0.0001	−1.82	6.66	0.20
right	Caudate	0.03	−0.01 to 0.07	1.37	−0.0009	−0.002 to 0.0004	−1.34	9.76	0.28
right	dACC	0.03	−0.001 to 0.07	1.92	−0.001	−0.002 to −0.00005*	−2.05	11.4	0.32
right	ITG	0.04	−0.003 to 0.08	1.84	−0.001	−0.002 to 0.0002	−1.74	12.00	0.33
right	Insula	0.04	0.001 to 0.07*	2.05	−0.001	−0.002 to −0.00002*	−2.00	3.19	0.09
right	MTG	0.05	0.01 to 0.09**	2.71	−0.002	−0.003 to −0.0004*	−2.56	12.40	0.34
right	Nacc	0.03	−0.008 to 0.06	1.52	−0.0009	−0.002 to 0.0002	−1.55	3.67	0.11
right	OFC	0.04	0.005 to 0.08*	2.24	−0.001	−0.002 to −0.0001*	−2.20	9.83	0.28
right	ParsO	0.05	0.02 to 0.09**	2.75	−0.002	−0.003 to −0.0005**	−2.83	11.30	0.31
right	PCC	0.04	0.04 to 0.08*	2.17	−0.001	−0.002 to −0.0001*	−2.15	12.60	0.34
right	Putamen	0.04	0.008 to 0.08*	2.44	−0.001	−0.003 to −0.0003*	−2.43	5.65	0.17
right	rACC	0.04	0.007 to 0.08*	2.34	−0.001	−0.003 to −0.0002*	−2.31	9.10	0.26
right	SFG	0.04	0.006 to 0.08*	2.25	−0.001	−0.003 to −0.0002*	−2.24	15.00	0.38
right	TP	0.05	0.01 to 0.1*	2.43	−0.002	−0.003 to −0.0003*	−2.39	5.35	0.16
right	Thalamus	0.03	0.00003 to 0.06*	1.97	−0.001	−0.002 to −0.00005*	−2.07	6.43	0.19
left	Amygdala	0.04	0.008 to 0.08*	2.43	−0.001	−0.003 to −0.0002*	−2.39	9.67	0.28
left	Caudate	0.03	−0.01 to 0.07	1.36	−0.0009	−0.002 to 0.0005	−1.27	8.96	0.26
left	dACC	0.03	−0.007 to 0.06	1.59	−0.001	−0.002 to 0.0002	−1.68	11.20	0.31
left	ITG	0.04	−0.002 to 0.07	1.87	−0.001	−0.002 to 0.0001	−1.77	13.40	0.31
left	Insula	0.03	−0.008 to 0.06	1.52	−0.0009	−0.002 to 0.0002	−1.55	5.21	0.16
left	MTG	0.03	−0.008 to 0.07	1.53	−0.0009	−0.002 to 0.0003	−1.42	15.40	0.16
left	Nacc	0.03	−0.01 to 0.06	1.34	−0.0008	−0.002 to 0.0004	−1.33	3.73	0.11
left	OFC	0.05	0.008 to 0.08*	2.42	−0.001	−0.003 to −0.0002*	−2.34	11.80	0.32
left	ParsO	0.03	−0.007 to 0.07	1.61	−0.001	−0.002 to 0.0002	−1.60	13.50	0.36
left	PCC	0.05	0.01 to 0.08**	2.65	−0.002	−0.003 to −0.0004**	−2.65	14.20	0.37
left	Putamen	0.03	−0.002 to 0.06	1.83	−0.001	−0.002 to 0.00008	−1.83	6.09	0.18
left	rACC	0.03	−0.003 to 0.07	1.81	−0.001	−0.002 to 0.0001	−1.81	9.51	0.27
left	SFG	0.04	0.002 to 0.08*	2.06	−0.001	−0.003 to −0.00005*	−2.06	15.90	0.40
left	TP	0.03	−0.01 to 0.07	1.45	−0.001	−0.002 to 0.0004	−1.45	5.29	0.16
left	Thalamus	0.03	−0.0004 to 0.06	1.95	−0.001	−0.002 to −0.00001*	−1.99	5.18	0.16

In the single ROI data analysis, linear regression model was used with factors including daylength, squared daylength, age, sex, BMI, and scanner type. The *t* values (*t*) of each factor, *F* values, and adjusted *R*^2^ of the full models are also listed. dACC, Dorsal anterior cingulate cortex; rACC, rostral anterior cingulate cortex; Nacc, nucleus accumbens; ITG, inferior temporal gyrus; MTG, middle temporal gyrus; OFC, orbitofrontal cortex; PCC, posterior cingulate cortex; ParsO, pars opercularis; SFG, superior frontal gyrus; TP, temporal pole.

*p < 0.05,

**p < 0.01.

### Causal effect of daylength on brain MOR availability in rats

**Figure 4.**
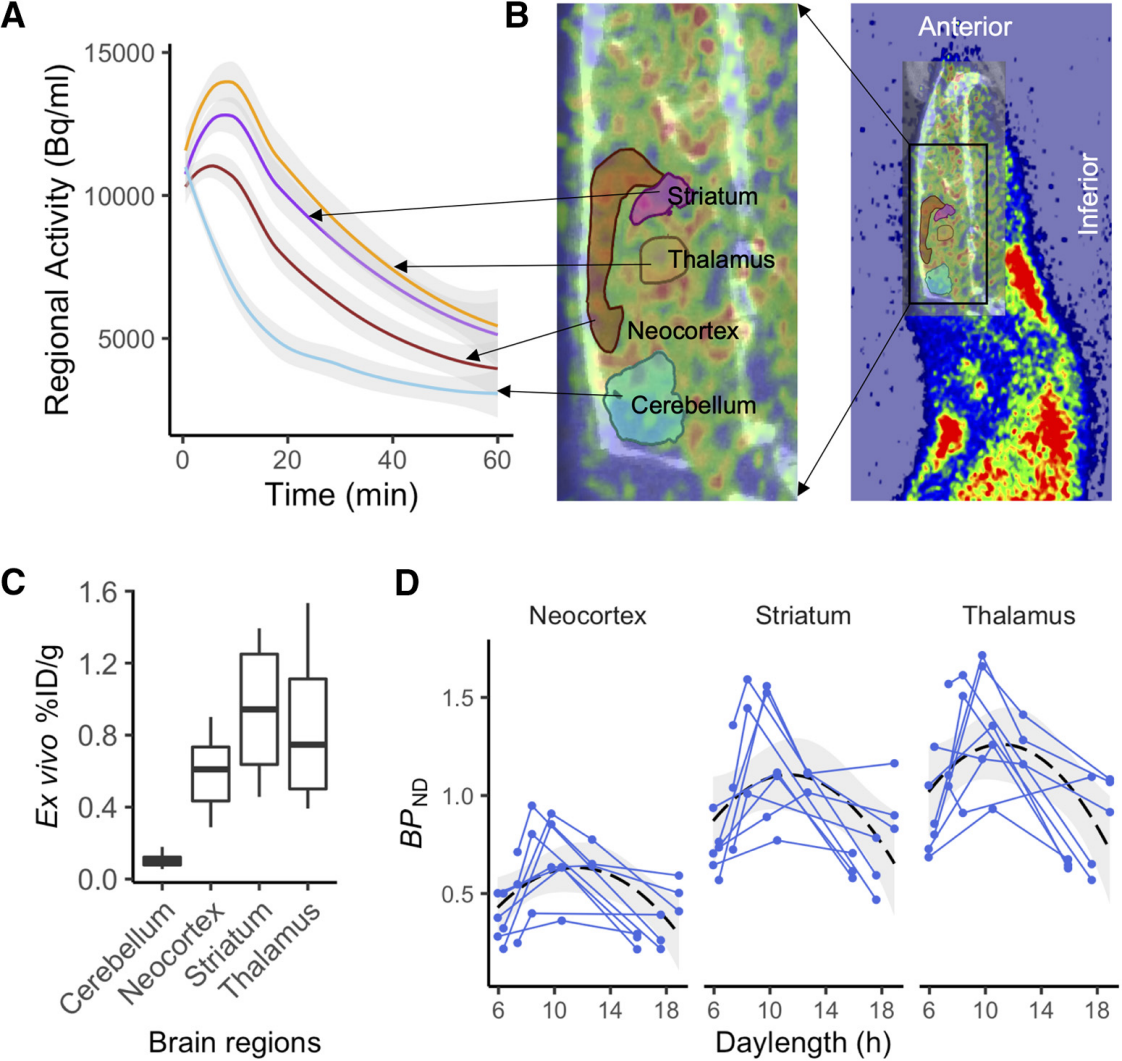
Within-animal changes in brain MOR availability as a function of daylength. ***A***, ***B***, Mean (with shaded area for 95% CI) time–activity curves for the reference region and the target regions (***A***) extracted using ROI template image overlaid on the PET and CT images (***B***). ***C***, *Ex vivo* biodistribution of [^11^C]carfentanil in brain regions of interests. ***D***, Individual animals are shown as separate lines. Black dashed line shows the least-squares (LS) regression curve for the polynomial model (*y* ∼ *x* + *x*^2^) predicting *BP*_ND_ across the whole sample. Shaded area shows the 95% CI for the LS curve.

Figure 4*A–C* demonstrates the regional time–activity curves and *ex vivo* gamma counting of [^11^C]carfentanil in the rat brain. To estimate the effects of daylength on MOR availability, we used a linear mixed-effects model with varying intercepts for each rat. Fixed-effect factors included daylength, squared daylength, age, sex, group, and the interaction effects between ROIs and all fixed-effect factors were included. When both experimental and control groups were included in the statistical model, and when all brain regions involved in the analysis, both daylength (β = 0.16; 95% CI, 0.035–0.28; *t* = 2.37) and squared daylength (β = −0.0067; 95% CI, −0.012 to −0.0017; *t* = −2.49) were significant predictors; the MOR availability and daylength had an inverted U-shaped relationship. Age, sex, or group did not influence MOR availability, and no interaction effects were found. Analysis excluding the control group, while using the same model excluding the fixed-effect factor group, gave similar results, with daylength (β = 0.16; 95% CI, 0.029–0.28; *t* = 2.24) and squared daylength (β = −0.0058; 95% CI, −0.011 to −0.00,057; *t* = −2.01) being significant predictors.

We also modeled regional *BP*_ND_ separately for the experimental group rats (within-animal design), using fixed-effect factors daylength and squared daylength and varying intercepts for each rat. Daylength and squared daylength were significant predictors for MOR *BP*_ND_ in all regions (Fig. 4*D*, Table 3). Including age and sex in the models did not improve the models, as evidenced by higher Akaike Information Criterion (AIC) values.

**Table 3.** Daylength and squared daylength have significant effects on brain regional MOR availability in rats in both simplified and full statistical models.

ROIs	Factors	β	95% CI	*t*	Marginal *R*^2^	AIC
Daylength and Squared daylength as fixed-effect factors						
Neocortex	Daylength	0.2	0.1 to 0.28	4.14	0.33	24.28
	Squared daylength	−0.008	−0.012 to −0.0045	−4.27		
Striatum	Daylength	0.25	0.11 to 0.38	3.57	0.26	48.16
	Squared daylength	−0.01	−0.017 to −0.005	−3.64		
Thalamus	Daylength	0.25	0.11 to 0.4	3.43	0.33	48.16
	Squared daylength	−0.01	−0.017 to −0.005	−3.75		
Full model						
Neocortex	Daylength	0.20	0.12 to 0.29	4.61	0.39	38.66
	Squared daylength	−0.008	−0.012 to −0.0047	−4.46		
Striatum	Daylength	0.25	0.11 to 0.39	3.49	0.30	60.17
	Squared daylength	−0.01	−0.017 to −0.004	−3.64		
Thalamus	Daylength	0.28	0.14 to 0.43	3.89	0.39	61.30
	Squared daylength	−0.01	−0.018 to −0.006	−3.79		

### Impact of seasonal cycle on weight gain in rats

To test whether the variable seasonal rhythm in the experimental group influenced weight gain, weight was analyzed using fixed-effect factors including age, sex, and group, and using rats as the random-effect factor. To see the effect of group on the rate of weight gain, we also included an interaction effect between group and age. Weight increased by aging (β = 0.96; 95% CI, 0.78–1.15; *t* = 10.02), and males had higher weights (β = 198; 95% CI, 117–216; *t* = 13.52). There was also an interaction between age and group (β = −0.31; 95% CI, −0.58 to −0.03), suggesting that simulated seasonal changes in daylength influenced the weight gain of rats. Separate analysis showed that weight gain as a function of aging was lower in the experimental group (β = 0.65; CI, 0.43–0.87; *t* = 6.07) than in the control group (β = 0.97; 95% CI, 0.81–1.17; *t* = 11.69).

### Impact of seasonal cycle on blood corticosterone levels in rats

Serum corticosterone levels were analyzed using fixed-effect factors including daylength, age, sex, and group, with rats as a random factor. Experimental group rats under seasonal cycle of daylength had a higher level of blood corticosterone (β = 44.93; 95% CI, 12.37–77.48; *t* = 2.71) compared with the control group. In addition to a group-level difference, daylength had a positive effect on corticosterone levels (β = 7.6; 95% CI, 2.22–12.98; *t* = 2.77). Aging also led to increased blood corticosterone levels (β = 1.55; 95% CI, 0.85–2.25; *t* = 4.32), and male rats had lower corticosterone levels (β = −39; 95% CI, −71.43 to −6.56; *t* = −2.36).

### Stress hormone corticosterone as a factor for brain MOR availability in rats

MOR binding in brain regions of interest was modeled using varying intercepts for rats and corticosterone level as the only fixed-effect factor. Corticosterone level was associated with MOR binding in the striatum (95% CI, −0.0033 to −0.000078; *t* = −2.03), but not in neocortex (95% CI, −0.0022 to 0.00025; *t* = −1.56) and thalamus (95% CI, −0.003 to 0.00027; *t* = −1.64). However, the effect in striatum was not significant after Bonferroni correction for multiple comparison.

## Discussion

Our main finding was that that seasonally varying daylength influences *in vivo* brain MOR availability in healthy humans and rats. Increasing daylength modulated brain MOR availability following an inverted U shape, with the highest MOR bindings observed in intermediate daylengths. The rat experiment established a causal effect of daylength on MOR availability, paralleling the quasi-experimental study in humans using a large database of historical PET imaging data. The most profound seasonal variation of MOR availability was found in large brain clusters including the amygdala, striatum, thalamus, cingulate cortex, orbitofrontal cortex, and superior frontal and temporal cortex. Thus, the present data show that the MOR system undergoes significant seasonal variation, which might be linked with seasonal variation in mood and sociability.

In high-latitude regions, daylight changes influence sleep quality, mood, and social behavior (Aronen et al., 2002; Lindblom et al., 2002; Hiltunen et al., 2011; Friborg et al., 2012). The prevalence of seasonal affective disorders or subsyndromes (time locked to short daylength) in the local region is remarkably high (Saarijärvi et al., 1999). The spring-like peak of MOR availability (Fig. 3*A*) parallels the seasonal variation in opioid-modulated emotional changes such as mood (Harmatz et al., 2000) and suicidality (Woo et al., 2012), particularly with prescription opioids (Davis et al., 2014), that peaks in the spring. Considering the impact of photoperiod on seasonal affective disorders (Magnusson and Boivin, 2003) as well as the intimate linkage between MOR signaling and social and affective functions (Nummenmaa and Tuominen, 2018; Lutz et al., 2020), the current study suggests that seasonal variation in MOR availability could be a potential brain mechanism contributing to seasonal affective changes.

Here, the human data revealed seasonal variation of MOR availability in brain regions linked with emotional and social processing, such as amygdala, cingulate cortex, and posterior superior temporal cortices (Nummenmaa and Tuominen, 2018). To our knowledge, this is the first *in vivo* evidence indicative of seasonal variation in MOR system in humans, and it fits with other types of evidence implying seasonal effects in neurotransmission. While there are no previous studies on seasonal patterns of brain MOR availability in humans, serotonin signaling shows seasonal patterns, although the findings are somewhat mixed. Serotonin transporter (SERT) availability is higher in fall and winter than in spring and summer in healthy humans, and the levels are negatively correlated with daily sunshine (Praschak-Rieder et al., 2008). Yet, another study found a positive correlation between 5-HT1A receptor availability and daylight in healthy males (Matheson et al., 2015). Patients with seasonal affective disorders (SADs) have higher SERT levels compared with healthy control subjects only in winter (Nørgaard et al., 2017), while light therapy leads to reduced SERT availability in patients with SADs (Tyrer et al., 2016). Monoamine oxidase A density in healthy humans (Spies et al., 2018) and striatal dopamine synthesis in patients with Parkinson's disease (Kaasinen et al., 2012) are also higher in fall and winter compared with spring and summer. Therefore, evidence on PET studies suggests seasonality effects on the brain across multiple neurotransmitter systems, and this is potentially driven by interactions between the specific systems (Tuominen et al., 2014).

The rat experiment demonstrated that daylength causally influenced brain MOR availability, paralleling the human data. These data are in line with the previously reported effects of photoperiod on MOR-dependent signaling in mammals (Nizielski et al., 1986; Lincoln et al., 1987; Meyza and Sotowska-Brochocka, 2006; Mukherjee and Haldar, 2016). The exact mechanism of the daylength-mediated alterations in brain MORs is, however, unclear. In mammals, neural responses to daylength are largely mediated by the superchiasmatic nucleus (SCN). The SCN generates endogenous neural signal, and adjusts the duration of this signal in accord with changes of the length of the solar day as is detected via the retinohypothalamic tract (Jacob et al., 1999; Schwartz et al., 2001). While MORs are most widely expressed in the subcortical and limbic brain regions, it is possible that daylength affects MOR signaling via the retinohypothalamic tract.

In rats, we also found that seasonally alternating daylength slowed weight gain and increased blood corticosterone levels in adult rats (compared with rats kept in a fixed 12 h light cycle), which is in line with previous findings (Deibel et al., 2014; Mariné-Casadó et al., 2018). Daylength-dependent changes in corticosterone levels may occur because of elevated stress caused by extremely long daylength, as supported by a positive effect of daylength on blood corticosterone levels in rats. In humans, exposure to extremely long daylength in summer also increases human blood cortisol levels (Vondrasová et al., 1997). Blood corticosterone levels have been linked with social stress and the activity of MOR signaling in mammals (Chong et al., 2006), where increased MOR avidity occurs along with lower cortisol level under stress. This is similarly supported by our finding that brain MOR availability in rats is negatively associated with blood corticosterone levels.

While the current study cannot disentangle the relation between corticosterone levels and MOR signaling in humans, the findings suggest that social and physiological stress could be linked with MOR availability. Although daylength is associated with improved mood (Harmatz et al., 2000; Magnusson and Boivin, 2003), cerebral MOR availability did not increase linearly with daylength but demonstrated an inverted U shape. Physiological or psychological stress may increase under extremely long daylength thus the results accord with the stress and brain function model conceptualizing an inverted U for benefits and costs of stress (Sapolsky, 2015). Further, this link between MOR signaling and stress is corroborated by the fact that opioid agonists and the partial agonist buprenorphine reduce the symptoms of anxiety and depression in rats (Falcon et al., 2016) as well as alleviate the effects of psychological stressors such as separation distress (Herman and Panksepp, 1978). In line with this, human molecular and functional imaging studies have found that high opioidergic tone suppresses hemodynamic brain responses to viewing distressing videos (Karjalainen et al., 2017).

### Limitations

While PET is the optimal approach to quantify *in vivo* brain MOR availability, certain limitations must be noted. The human study was based on historical data, where each subject was imaged only once, and quasi-experimental design (natural changes in daylight), as longitudinal multiscan studies would yield a significant radiation load. Contribution of other seasonal factors (e.g., temperature) cannot, however, be ruled out in the human data, whereas we verified the causal effect of daylength on brain MOR availability using animal experiments. Furthermore, daylength was estimated using astronomical data without considering day-to-day (e.g., cloud cover) and individual variation (e.g., time spent outside) in light exposure. Nevertheless, we had a large sample, and the results paralleled those from the experiment performed in rats, albeit with smaller effect size. The human data were also sampled from different projects and scanners, which were corrected for potential scanner-related biases in the analyses. Technically, while [^11^C]carfentanil *BP*_ND_ in baseline condition is proportional to MOR density, the exact contributions of MOR density, receptor affinity, and baseline occupancy by endogenous opioids cannot be assessed in a single measurement (Mintun et al., 1984; Henriksen and Willoch, 2008), and these components cannot be differentiated in a single scan. [^11^C]carfentanil is an agonist tracer preferably binding MORs in the high-affinity state (Henriksen and Willoch, 2008; Cumming et al., 2019) and has low test–retest variability in PET imaging (Hirvonen et al., 2009). Although endogenous opioids compete for binding sites with [^11^C]carfentanil (Quelch et al., 2014; Saanijoki et al., 2018), the basal opioid concentrations are often very low, at least in rats (Maidment et al., 1989). Accordingly, [^11^C]carfentanil *BP*_ND_ in baseline likely reflects density of MORs. Also, the human database was compiled from historical subjects spanning multiple projects, where relevant behavioral and self-report measures on mood and social behavior were not systematically collected. The rat experiment simulated causal seasonal effects of daylength on MOR availability, yet we decided against behavioral measures as the stress caused by repeated behavioral tests would likely confound seasonality-dependent MOR variation. Instead, we measured stress hormones and monitored weight gain as physiological markers. These design features, however, preclude us from making strong claims regarding the causal role of the seasonal variation in MOR and socioemotional behavior. Finally, current findings based on human data should be interpreted while keeping in mind the large magnitude of local photoperiodic variation, which should be cautiously interpolated into regions with lower latitudes.

### Conclusions

We conclude that seasonal variation in daylength influences brain MOR availability, following an inverted U-shaped curve in both humans and rats. Given the intimate links between MOR signaling and socioemotional behavior (Becker et al., 2014; Lutz et al., 2020), these results suggest that the MOR system might underlie the seasonal variation in human mood and social behavior (Harmatz et al., 2000; Lam and Levitan, 2000) and imply that MOR might be a feasible target for treating seasonal affective disorders.
